# Pental

**Published:** 1895-09

**Authors:** 


### SELECTIONS.

### Pental.

Mr. T. E. Constant read, a paper at the Odontological Society recently on "The Production of General Anæsthesia for Dental Operations by Means of Pental." He remarked that pental is a new drug only as regards its name and method of preparation; it is, in fact, the amylene of Snow. The manufacturer, C. A. F. Kahlbaum, of Berlin, claims for it a definite and unvarying chemical composition and freedom from all impurities. Pental, or isoamylene, $(CH_3)_2C.CH.CH_3$, is a colorless liquid of low specific gravity, having a constant boiling-point of 38° C. It is obtained from amylene hydrate by heating with acids. It is insoluable in water, but mixes with chloroform, ether, and alcohol. It is extremely volatile and inflammable. It has a peculiar and somewhat disagreeable odor, but is so little irritating that the pure vapor can be inhaled without the slightest discomfort. In his early experiments, Mr. Constant endeavored to induce anæsthesia by the open method,—that is, by pouring the drug upon a piece of lint lying in a cone-shaped holder; but this he found unsatisfactory owing to the waste of time and the quantity used. Subsequently he devised an apparatus somewhat on the principle of the Ormesby inhaler. In the majority of the cases the following phenomena were noticed: Almost immediately the pental was inhaled there were slight flushing of the face and quickening of the pulse, the increasing frequency being unaccompauied by any diminution of force. Respiration, if quiet at the commencement of the inhalation, became deep and rapid when the handle of the inhaler was turned full on, but became quieter when it was turned off. The eyes, if closed, opened as the patient became anæsthetized, and had a fixed and staring look. The conjunctiva reflex was rarely lost, although in some cases it was absent after four or five inspirations. In a few instances there was profuse perspiration after thirty seconds. The duration of anæsthesia produced was on the average of one and a half minutes, but varied greatly with the patient and with the character of the respiration, never having been less than a minute or more than three; there was no muscular relaxation. Occasisnally the patient's eyes would follow the movements of the operator; otherwise the patient remained quite still. In about five of the one hundred and forty cases the patients declared they were perfectly concious throughout, but felt no pain and had no desire to move. There were no after-effects immediate or remote, in any one case where it was administered in this way. However, he had cases where dangerous symptoms arose, and recently three fatal cases had been reported.

In conclusion, Mr. Constant said that whatever may be the safety of pental as compared with chloroform, there can be no doubt that it is more dangerous than nitrous oxide, and the last-named agent should invaraiably be chosen for breif operations. Nevertheless, he was of opinion that those of the medical profession who are in the habit of administering chloroform for operations where nitrous oxide, or nitrous oxide and ether, conld be used equally well should give the preference to pental, because, even if it be no safer than chloroforn (and personally the writer believes it to be far more so), the ease of administration, the certainty of its action, the rapidity of recovery after its use, and the entire absence of after-affects entitle it to a claim upon the consideration of those who deem chloroform a justifiable anæsthetic in dental surgery.—*Lancet*, April 28, 1894.

